# Commentary: Calcium arrhythmogenicity of Purkinje fibers: importance of the animal model

**DOI:** 10.3389/fphys.2026.1970067

**Published:** 2026-10-07

**Authors:** Sebastien Chaigne

**Affiliations:** 1 IHU Liryc, INSERM, U1045, CRCTB, University Bordeaux, Bordeaux, France; 2 CHU de Bordeaux, Cardiology, INSERM, U1045, CRCTB, Bordeaux, France

**Keywords:** aging, animal models, Ca^2+^ homeostasis, Purkinje fiber cells, TRPV4

The recent mini-review by Stuyvers (2025) reports the distinct Ca^2+^ handling properties of Purkinje fiber (PF) cells that contribute to post-infarction ventricular arrhythmogenesis. This review provides a valuable overview of species-dependent differences in PF cell physiology and also discusses the limitations related to murine models. These considerations are essential for a better understanding of ventricular arrhythmias and for improving translational research. Based on this, we would like to highlight a potential Ca^2+^ entry pathway that may contribute to Ca^2+^ dysregulation and arrhythmogenic vulnerability in PF cells: the transient receptor potential vanilloid 4 (TRPV4) channel. TRPV4 is a broadly expressed, non-selective Ca^2+^-permeable cation channel found in several cardiovascular cell types (Hof et al., 2019) suggesting a potential contribution to Ca^2+^-dependent regulation of cardiac electrophysiology. Although TRPV4 has not yet been directly characterized in PF cells, its expression and functional activity have been demonstrated in ventricular cardiomyocytes (Jones et al., 2019; Adapala et al., 2020; Peana et al., 2022; Chaigne et al., 2023). In mouse ventricular cardiomyocytes, TRPV4 activity has been shown to modulate electrical properties, including action potential duration and excitability (Chaigne et al., 2021). Pharmacological TRPV4 activation prolongs action potential duration through a Ca^2+^-dependent mechanism, whereas this effect is reduced by TRPV4 inhibition and absent in TRPV4-knockout mouse models (Chaigne et al., 2021). Additionally, TRPV4 expression and activity in mice are modulated by aging, osmotic stress; hypertrophic remodeling, and ischemic injury (Veteto et al., 2020; Peana et al., 2022; Chaigne et al., 2023; Stuyvers, 2025); emphasizing the need to consider both species and age when choosing appropriate experimental models. These aspects may be particularly relevant to ventricular arrhythmias in elderly patients, where aging, ischemic remodeling, and altered myocardial mechanical properties influence TRPV4-dependent Ca^2+^ regulation. In PF cells, abnormal Ca^2+^ handling, characterized by sarcoplasmic reticulum Ca^2+^ overload, NCX-dependent depolarizing currents, and altered Ca^2+^-dependent regulation of electrophysiological properties, represents a key substrate for triggered activity. TRPV4-mediated Ca^2+^ influx could contribute to this arrhythmogenic cascade by increasing cytosolic Ca^2+^ load, promoting SR Ca^2+^ overload, and enhancing NCX-dependent delayed afterdepolarizations (DADs) (cf. **Figure 1**). Although this hypothesis remains to be experimentally validated in PF cells, it provides a mechanistic framework linking extracellular stress signals to intracellular Ca^2+^-dependent arrhythmogenic pathways.

**Figure 1:**
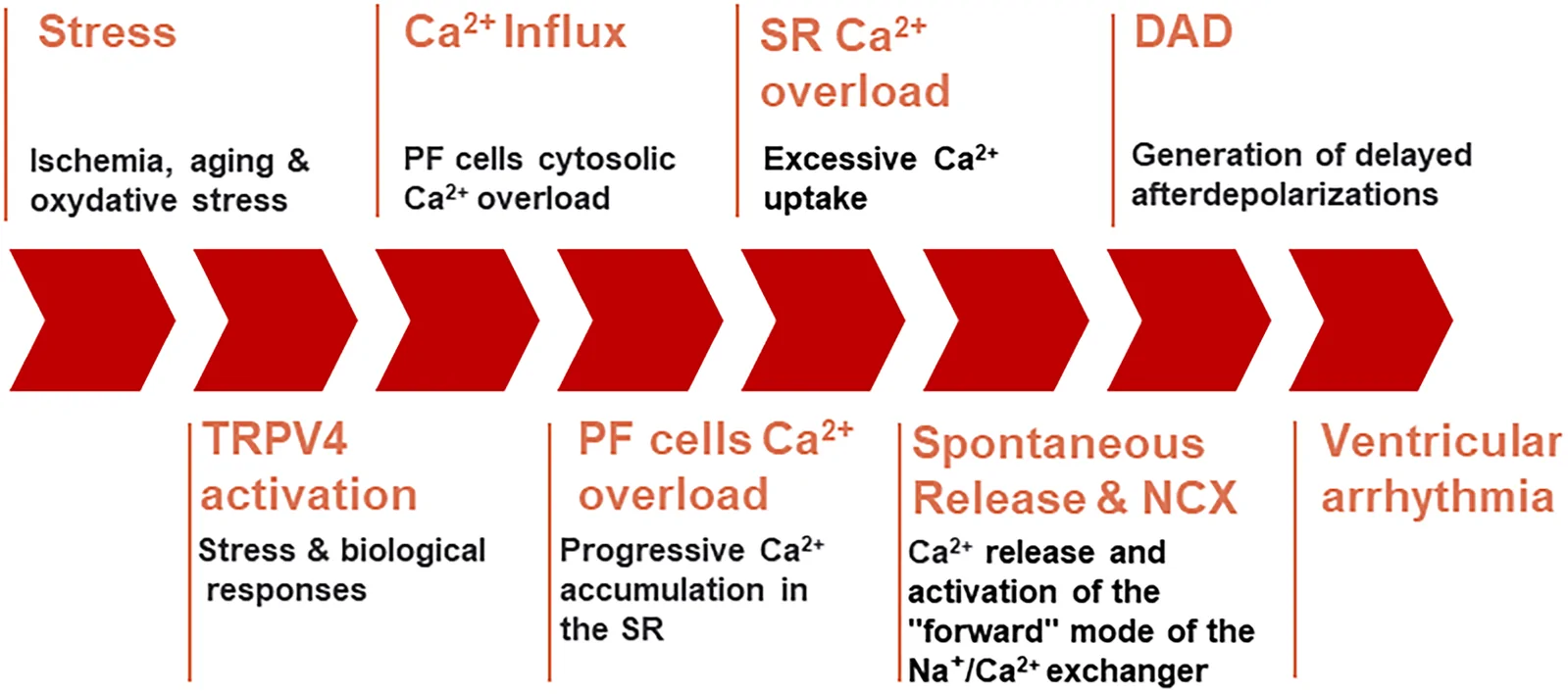
Does TRPV4 contribute to calcium-driven arrhythmogenesis in Purkinje fibers? Stress may activate TRPV4 channels, causing cytosolic and then sarcoplasmic reticulum (SR) Ca^2+^ overload. This triggers spontaneous SR Ca^2+^ release and forward mode Na^+^/Ca^2+^ exchanger (NCX) activity, generating a depolarizing current that may produce delayed after depolarizations (DADs) and, ultimately, ventricular arrhythmias.

Furthermore, the mechanosensitive and stress-responsive properties of TRPV4 (Veteto et al., 2020) make this channel a plausible contributor to abnormal Ca^2+^ handling observed in PF cells after myocardial infarction, a cardiac phenotype characterized by increased mechanical stress, inflammation, and oxidative stress. A key point is that PF cell Ca^2+^ handling differs substantially across species, and murine PF cells do not fully recapitulate the physiology of PF cells from large mammals. Importantly, these species differences are likely not restricted to Ca^2+^ handling proteins themselves but may also reflect variations in the mechanisms that control Ca^2+^ entry and in how cells respond to environmental and stress conditions.

Therefore, translating findings from mouse models to human arrhythmogenic conditions remains challenging, especially for the TRPV4 channel, as its expression and regulation may differ between animal models. This knowledge gap offers an opportunity to explore whether TRPV4 participates in the regulation of Ca^2+^ handling in large-mammal PF cells, thereby extending the species-specific framework proposed by the author.

The availability of selective TRPV4 antagonists that have reached clinical evaluation (Goyal et al., 2019) further supports the translational interest of investigating this channel in large-mammal Purkinje models and may identify a potential treatment for post-infarction ventricular arrhythmias. In this context, TRPV4 may represent an illustrative example of how large-mammal Purkinje models could be essential to uncover clinically relevant mechanisms underlying PF cell arrhythmogenesis.

Recent methodological advances, including AI-based identification of cardiac PF cells isolated from whole adult sheep hearts, offer new strategies to investigate arrhythmogenic mechanisms in large-mammal Purkinje models with greater translational relevance (Chaigne et al., 2025). Such approaches may improve the efficiency and reproducibility of PF-cell identification and characterization, facilitating biophysical studies of ion channel function in large-mammal Purkinje fibers.

We believe this concept complements, rather than challenges, the framework proposed by Stuyvers and raises the possibility that TRPV4 and other Ca^2+-^ permeable TRP channels (Hof et al., 2019 and Kulkarni et al., 2025), as key regulators of Ca^2+^ influx and cellular stress responses, merit further investigation in large mammal Purkinje models of ischemic arrhythmias.
