# Correction: Commentary: Calcium arrhythmogenicity of Purkinje fibers: importance of the animal model

**DOI:** 10.3389/fphys.2026.2025326

**Published:** 2026-10-28

**Authors:** Sebastien Chaigne

**Affiliations:** 1 IHU Liryc, INSERM, U1045, CRCTB, University Bordeaux, Bordeaux, France; 2 CHU de Bordeaux, Cardiology, INSERM, U1045, CRCTB, Bordeaux, France

**Keywords:** aging, animal models, Ca2+ homeostasis, Purkinje fiber cells, TRPV4

There was a mistake in the caption of **Figure 1** as published

“FIGURE 1 Does TRPV4 contribute to calcium-driven arrhythmogenesis in Purkinje fibers? Stress may activates TRPV4 channels, causing cytosolic and then sarcoplasmic reticulum (SR) Ca2+ overload. This triggers spontaneous SR Ca2+ release and forward-mode Na+/Ca2+ exchanger (NCX) activity, generating a depolarizing current that (MODIFICATION HERE) 'may' produces delayed afterdepolarizations (DADs) and, ultimately, ventricular arrhythmia.”

The corrected caption of **Figure 1** appears below.

“FIGURE 1 Does TRPV4 contribute to calcium-driven arrhythmogenesis in Purkinje fibers? Stress may activate TRPV4 channels, causing cytosolic and then sarcoplasmic reticulum (SR) Ca^2+^ overload. This triggers spontaneous SR Ca^2+^ release and forward-mode Na^+^/Ca^2+^ exchanger (NCX) activity, generating a depolarizing current that may produce delayed afterdepolarizations (DADs) and, ultimately, ventricular arrhythmias.”

Superscript numbers were erroneously included in the text, and the formatting of some references was incorrect.

A correction has been made to the first paragraph:

“Although TRPV4 has not yet been directly characterized in PF cells, its expression and functional activity have been demonstrated in ventricular cardiomyocytes (Jones et al., 2019; Adapala et al., 2020; Peana et al., 2022; Chaigne et al., 2023).”

“Additionally, TRPV4 expression and activity in mice aremodulated by aging, osmotic stress; hypertrophic remodeling, and ischemic injury (Veteto et al., 2020; Peana et al., 2022; Chaigne et al., 2023; Stuyvers, 2025); emphasizing the need to consider both species and age when choosing appropriate experimental models.”

A correction has been made to the last paragraph:

“We believe this concept complements, rather than challenges, the framework proposed by Stuyvers and raises the possibility that TRPV4 and other Ca^2+^-permeable TRP channels (Hof et al., 2019 and Kulkarni et al., 2025), as key regulators of Ca^2+^ influx and cellular stress responses, merit further investigation in large-mammal Purkinje models of ischemic arrhythmias.”

The original version of this article has been updated.

